# Human whole mitochondrial genome sequencing and analysis: optimization of the experimental workflow

**DOI:** 10.3325/cmj.2022.63.224

**Published:** 2022-06

**Authors:** Viktorija Sukser, Marina Korolija, Ivana Račić, Sara Rožić, Lucija Barbarić

**Affiliations:** Department of Biology and Fibers, Forensic Science Centre “Ivan Vučetić,” Zagreb, Croatia

## Abstract

**Aim:**

To evaluate critical steps in Illumina® Human mtDNA Genome assay: target enrichment, limited-cycle PCR, and library normalization, in order to optimize the protocol for analysis of whole mitochondrial genomes from human reference samples.

**Methods:**

Three long-range high-fidelity DNA polymerases (Platinum^TM^ PCR SuperMix High Fidelity, LA Taq® Hot Start, and PrimeSTAR® GXL) were tested for their performance in the amplification of mtDNA fragments. Sequencing results of ten samples, as well as negative controls, which underwent library preparation with 12 and 15 cycles in limited-cycle PCR were compared. Additionally, two library normalization methods were compared: bead-based normalization vs quantification and individual normalization.

**Results:**

PrimeSTAR® GXL performed best for mitochondrial DNA enrichment. Increment of amplification cycles to 15 in limited-cycle PCR step did not affect either the sequencing process or variant calling. Library quantification combined with individual library-by-library dilution outperformed bead-based normalization.

**Conclusion:**

Optimizations described herein provide beneficial insights for laboratories aiming at implementation and/or advancement of similar massively parallel sequencing workflows (eg, small genomes, PCR amplicons, and plasmids).

Library preparation in massively parallel sequencing (MPS) protocols is a sensitive process, usually consisting of multiple elaborate steps. To ensure high quality of sequencing results, it is important to optimize library preparation according to characteristics of a particular target molecule. There are several critical aspects of Illumina® Human mtDNA Genome (1) assay for analysis of whole mitochondrial DNA (mtDNA) on MiSeq® instrument: initial enrichment of the target molecule (achieved, in this case, by long-range PCR); PCR step wherein index-adapter oligonucleotides are added and libraries are amplified (termed “limited-cycle PCR” step); and normalization of libraries prior to their pooling for sequencing.

In this study, we aimed to test three long-range high-fidelity DNA polymerases for their performance in amplification of mtDNA fragments, in order to determine the one best suited for Illumina® assay, in which mitochondrial genomes are amplified in two large fragments (sizes 9.1 kb and 11.2 kb) (Supplementary Figure 1(Supplementary Figure 1)).

Various optimizations and evaluations have been previously published (2-6), but, to our knowledge, none of them assessed the impact of increasing the number of amplification cycles in limited-cycle PCR. Therefore, we also aimed to test and observe how sequencing results were affected by this step and whether there were any adverse effects that would impact variant calling.

Lastly, we aimed to compare two library normalization methods: bead-based normalization vs quantification and individual normalization. Nextera® XT Library Prep Kit (Illumina, San Diego, CA, USA) has been known to produce uneven read depth profiles (2-4,6-8). Therefore, a great risk in this protocol is potential loss of sequence information in regions that achieve very low read depth (ie, too low for analysis and subsequent variant calling), or possibly receive no reads at all. The choice of library normalization method may affect this through distribution of reads among the sequenced libraries. So, a method that provides more uniform distribution of reads would be preferable, which we hypothesized would be the method of quantification and individual normalization rather than bead-based normalization.

## MATERIAL AND METHODS

### DNA extraction

The samples were collected during a 3-year time interval (spanning 2016-2018) within Biology and Fibers Department, Forensic Science Centre “Ivan Vučetić,” Zagreb, Croatia. Samples of buccal epithelium and blood were collected from laboratory employees and volunteers (50 in total), who gave detailed informed consent to participate in the study. Buccal epithelia samples were collected on Whatman^TM^ Sterile Omniswab (GE Healthcare, UK), while blood samples were collected on Whatman^TM^ FTA^TM^ Classic Cards. All samples were air-dried prior to DNA extraction. DNA was extracted from buccal swabs using EZ1® DNA Investigator® Kit (Qiagen, Hilden, Germany) (9): pre-treatment protocol included adding 290 μL of buffer G2 and 10 μL of proteinase K, followed by 2-hour incubation at 56 °C. Samples then underwent automated purification on EZ1® Advanced XL instrument (Qiagen), by using “Trace Protocol” with elution in TE buffer, resulting in final volume of 100 μL. From dried blood samples on FTA cards, DNA was extracted and purified with QIAamp MinElute columns from QIAamp® DNA Micro Kit (Qiagen) (10), following the manufacturer’s protocol for “isolation of genomic DNA from dried blood spots” (final sample volume was 50 μL). Purified DNA isolates were quantified on Qubit^TM^ 3.0 Fluorometer with Qubit^TM^ dsDNA High Sensitivity kit (Thermo Fisher Scientific, Waltham, MA, USA), according to the manufacturer’s instructions (11).

### Target enrichment

Three long-range, high-fidelity DNA polymerases were tested for the target enrichment step: Platinum^TM^ PCR SuperMix High Fidelity (Thermo Fisher Scientific), LA Taq® Hot Start (TaKaRa, Kusatsu, Japan), and PrimeSTAR® GXL (TaKaRa) (12-14). Experiments were organized sequentially, as shown in Table 1, with thermal cycling conditions detailed in Table 2. First, the performance of all three DNA polymerases was assessed by the amplification of 11.2 kb mtDNA fragment from the same buccal swab sample. Second, two better performing DNA polymerases were further compared for the amplification of 11.2 kb fragment. Onwards, PrimeSTAR® GXL was solely assessed for the amplification of both mtDNA fragments, 9.1 kb and 11.2 kb, from both types of samples – buccal swabs and blood. PCR products were visualized and evaluated via agarose gel electrophoresis (Supplementary Table 1(Supplementary Table 1)).

**Table 1:** Layout of experiments to test the performance of three long-range high-fidelity DNA polymerases: Platinum^TM^ PCR SuperMix High Fidelity (Platinum), LA Taq® Hot Start polymerase (LA) and PrimeSTAR® GXL polymerase (GXL)

Test stage	Polymerase	Amplicon (kb)	Sample type	Input genomic DNA (ng)*	Amplification cycles (N)
**1**	Platinum	11.2	Buccal	1.5, 3, 6	30
	LA				
	GXL				
**2**	LA	11.2	Buccal	0.25, 0.5, 1, 2	15, 18, 21, 24, 27, 30
	GXL				
**3**	GXL	9.1	Buccal	0.25, 0.5, 1, 2	30
**4**	GXL	9.1	Buccal & blood	1	25, 30
**Final**	GXL	9.1 & 11.2	Buccal & blood	1	25

*Amount of DNA in reaction volume 12.75 μL (Platinum) and 12.5 μL (LA and GXL). Reagent volumes were adjusted proportionately, according to volumes listed in manufacturers’ instructions (12-14).

**Table 2:** Thermal cycling conditions applied for Platinum^TM^ PCR SuperMix High Fidelity (Platinum), LA Taq® Hot Start polymerase (LA), and PrimeSTAR® GXL polymerase (GXL)

Platinum*			LA^†^			GXL^‡^		
**11.2 kb**			**11.2 kb**			**11.2 (2-step)**		
94 °C	2 min		94 °C	5 min		98 °C	10 s	× N
94 °C	20 s	× N	98 °C	15 s	× N	68 °C	10 min	
59 °C	20 s		68 °C	10 s				
68 °C	11 min 12 s			slow ramp 0.2 °C/s to 60 °C		9.1 (3-step)		
68 °C	10 min		60 °C	15 s		98 °C	10 s	× N
			68 °C	11 min		55 °C^§^	15 s	
			72 °C	10 min		68 °C	9 min 6 s	

*As in manufacturer's instructions (12).

†As in Illumina protocol (1).

‡As in manufacturer's instructions (14).

§Annealing temperature later increased to 60 °C.

### Library preparation

Samples underwent long-range PCR with PrimeSTAR® GXL DNA polymerase, by using the established optimized conditions for 9.1 kb fragment: 25 × (98 °C 10 s, 60 °C 15 s, 68 °C 9 min, 6 s), and for 11.2 kb fragment: 25 × (98 °C 10 s, 68 °C 10 min). Both mtDNA amplicons were then quantified with Qubit^TM^ dsDNA High Sensitivity kit on Qubit^TM^ 3.0 Fluorometer (Thermo Fisher Scientific) and individually normalized to the concentration of 0.2 ng/μL, as described in the Illumina® protocol (1). Both mtDNA amplicons (9.1 kb and 11.2 kb) were then pooled for each sample, wherefrom the volume of 5 μL was taken for library preparation (ie, total input of 1 ng). Nextera® XT Library Prep Kit (Illumina) was used according to the manufacturer’s instructions (1). Negative controls (NCs), which consisted of reagent blanks, were introduced at three stages of the workflow: DNA extraction (NC-EX), target enrichment (NC-PCR), and library preparation (NC-LIB). All NCs underwent the same library preparation procedure as the tested samples.

Exceptions from the manufacturer’s library preparation protocol were introduced in the step where index-adapters are added, which is termed “limited-cycle PCR.” Libraries were prepared with 12 cycles (as per protocol) and 15 cycles. Sequencing results from 10 samples of buccal epithelia that underwent both original and modified protocol were later compared, along with negative controls sequenced in respective runs.

Libraries were purified with Agencourt AMPure XP magnetic beads (Beckman Coulter, Brea, CA, USA). Afterwards, another departure from protocol was introduced: libraries underwent either bead-based normalization (reagents provided in the library prep kit), or quantification with LabChip® DNA High Sensitivity Assay (15) on LabChip® GX Touch HT (PerkinElmer, Waltham, MA, USA) plus individual dilution of libraries. Of all the runs performed in the course of this study, but also in parallel studies (not disclosed herein), six sequencing runs containing identical or approximately the same number of samples (both buccal epithelia and blood) were singled out for comparison, in order to assess the impacts of two different library normalization methods (three runs for each method).

### Sequencing

Normalized libraries were pooled, denatured, and diluted as described in Illumina® protocol (16), with a 5% spike-in of PhiX Sequencing Control v. 3 (Illumina). Paired-end sequencing was performed on Illumina® MiSeq FGx^TM^ instrument by using MiSeq® Reagent Kit v.2, 300 cycles (2 × 151 bp).

### Data analysis and statistical calculations

Sequencing quality metrics were reviewed in Illumina® Sequencing Analysis Viewer v.1.11.1 software. FASTQ files generated on instrument by MiSeq® Reporter were extracted and uploaded to Illumina® BaseSpace® Sequence Hub online platform, where they were analyzed by BaseSpace® mtDNA Variant Processor v.1.0.0 App (17). The latter used rCRS genome (18,19) for alignment, and analysis thresholds previously established by internal evaluation (8), most notably: minimum of 220 reads for variant calling, analysis threshold of 3%, and interpretation threshold of 6%. NCs were analyzed at lower thresholds (read depth 2, analysis and interpretation thresholds at 0.1%) (8) in order to assess all detected signals. Read depth of detected signals in NCs was assessed, as well as total number of positions with detected signals. Sequence variants and read depth profiles were visually inspected in BaseSpace® mtDNA Variant Analyzer v.1.0.0 App, as well as in IGV browser (20,21). Variant reports in Excel format, as well as VCF files, were exported from BaseSpace®. Microsoft Office Excel was used for further review of variant reports, variant confirmation, and comparison of results. Statistical calculations (*t* test: paired two-sample for means, and *t* test: two-sample assuming unequal variances) were performed with Data Analysis add-in, Microsoft Office Excel.

## RESULTS

### Long-range PCR for mtDNA enrichment

Initially, Platinum PCR SuperMix performed very poorly (Supplementary Figure 2A(Supplementary Figure 2)), while LA Taq Hot Start and PrimeSTAR GXL both yielded bands of expected size – 11.2 kb (Supplementary Figure 2B and 2C, respectively(Supplementary Figure 2)). In the second stage, optimal DNA input and the number of amplification cycles were tested (Supplementary Figure 3(Supplementary Figure 3)). Despite visually similar results, PrimeSTAR GXL produced slightly clearer and better-defined bands for 11.2 kb amplicon than LA Taq Hot Start. In the third stage, optimal genomic DNA input was determined for PrimeSTAR GXL, equaling 1 ng of genomic DNA in 12.5 μL reaction volume (Supplementary Figures 4(Supplementary Figure 4) and 5(Supplementary Figure 5)). The optimal number of amplification cycles was 25, with annealing temperature increased from 55 °C to 60 °C (Supplementary Figure 5(Supplementary Figure 5)). To confirm the final thermal cycling conditions (Table 1), both mtDNA fragments were amplified in different samples and sample types (Supplementary Figure 6(Supplementary Figure 6)).

### Evaluation of limited-cycle PCR

Libraries of ten samples (buccal epithelia) were used for evaluation, and were sequenced in four runs: two for 12-cycle libraries and two for 15-cycle libraries. All four runs were of good quality: cluster density was higher for 15-cycle libraries (1533 and 1383 K/mm^2^ vs 864 and 955 K/mm^2^), but “clusters PF” was >84% in all runs and “% bases ≥Q30” was >91%, also in all runs. Molar concentrations of libraries amplified with 15 cycles showed substantial increase – approximately 3 to 11 times – as opposed to libraries that underwent 12-cycle PCR (Table 3). LabChip electropherograms showed larger quantities of library fragments, along with improved distribution with the characteristic “hill” (Supplementary Figure 7(Supplementary Figure 7)). All variant calls, ie, mitochondrial haplotypes, were concordant between 12-cycle and 15-cycle libraries of corresponding samples, including occurrences of point heteroplasmies (PHPs) in 7 out of 10 samples. Percentages of minor alleles detected in PHPs (Supplementary Table 2(Supplementary Table 2)) were consistent between 12-cycle and 15-cycle libraries, and differences were not significant (*t* test, paired; *P* > 0.05). Average read depth (Table 2) was greater for 15-cycle libraries; however, this is a consequence of higher cluster density in corresponding runs, and not connected directly to limited-cycle PCR.

**Table 3:** Molar concentrations, amount of reads (% reads identified), and average read depth (reads) of libraries prepared with 12-cycle and 15-cycle amplification in limited-cycle PCR step. Libraries were quantified by using LabChip® DNA High Sensitivity Assay on LabChip® GX Touch HT

	12-cycle library			15-cycle library		
Sample ID	Molarity (nmol/L)	% reads identified	Average read depth	Molarity (nmol/L)	% reads identified	Average read depth
**MW-004**	12.8	2.2	5216	43.77	1.9	5882
**MW-005**	6.5	1.5	3734	45.23	1.6	5270
**MW-019**	6.8	2.1	5222	36.17	2.9	9895
**MW-020**	8.9	2.1	6445	34.85	4.1	13850
**MW-026**	4.1	1.1	4083	46.31	1.5	5523
**MW-051**	4.8	1.9	4106	46.67	2.1	6742
**MW-152**	5.9	2.8	4272	46.02	3.9	12295
**MW-236**	11.4	2.3	5611	47.34	2.2	7196
**MW-290**	5.1	2.0	5718	37.73	2.1	7080
**MW-302**	7.9	2.0	5611	47.60	2.5	8423

Negative controls (NC-EX, NC-PCR, and NC-LIB) were analyzed as previously described (8) to assess the level of noise and exogenous signals detected in sequencing. In total, 8 and 6 negative controls were analyzed for 12 and 15 cycles, respectively. Cumulatively, an average of 7370 positions with reads were detected in 15-cycle NCs, which is higher than the average of 5669 detected in 12-cycle NCs (Supplementary Table 3(Supplementary Table 3)). Nonetheless, average read depth was only slightly elevated (6 reads and 4 reads for 15-cycle and 12-cycle NCs, respectively), while maximum read depth detected in any NC was well below the established minimum read depth threshold of 220 reads (8).

Noise in samples was evaluated by analyzing signals of alternative bases (different than haplotypes, excluding positions with PHP): average read depth of alternative signals was 44 reads for 12-cycle libraries, and 52 reads for 15-cycle libraries. In both cases, there were signals detected with >220 reads, however, all were below the 3% analysis threshold (8) and exhibited poor strand balance and/or low quality score. Therefore, these signals were filtered out and excluded from final variant calling, and did not affect interpretation of true variants.

### Comparison of library normalization methods

Two library normalization methods were compared: magnetic beads-based normalization against quantification of libraries followed by individual normalization. Three sequencing runs were compared for each method, and quality metrics for all showed good quality (Table 4). Values of “% reads identified” were close to expected values (calculated from the number of samples per run). Coefficients of variation showed that bead-based normalization introduced slightly greater variation to the distribution of reads per library (Table 4, Supplementary Figure 8(Supplementary Figure 8)). However, differences between the two sets were not significant (*t* test, unequal variances; *P* > 0.05).

**Table 4:** Quality metrics for sequencing runs compared to assess library normalization methods. In runs 1-3 libraries were normalized with magnetic beads included in the Nextera® XT library prep kit, while in runs 4-6 individual dilution was applied after quantification with LabChip® DNA High Sensitivity Assay on LabChip® GX Touch HT

Run	No. of samples	Normalization method	Cluster density (K/mm^2^)	Clusters PF (%)	% bases≥Q30	% reads identified ± standard deviation (expected %)	% CV
1	24	Bead-based	939	94.1	93.0	4.0 ± 1.3 (4.2)	32.1
2	24		491	97.8	97.1	3.9 ± 1.8 (4.2)	47.1
3	24		1062	91.5	93.8	4.1 ± 1.6 (4.2)	40.0
4	24	LabChip-based	864	93.6	93.4	4.0 ± 0.6 (4.2)	16.1
5	28		948	93.7	96.1	3.7 ± 0.7 (3.6)	19.4
6	24		551	96.0	95.7	4.1 ± 1.7 (4.2)	41.7

## DISCUSSION

Key features evaluated in the process of DNA polymerases testing were clearly defined, specific bands of expected size, without smears or non-specific products while visualized by gel-electrophoresis. This empirical approach singled out PrimeSTAR GXL as best suited for the amplification of whole mtDNA in two large fragments, since it provided good balance between yield and specificity for both buccal epithelia and blood sample types. In the end, thermal cycling conditions for 9.1 kb consisted of 3-step PCR, with annealing temperature increased to 60 °C, while 2-step PCR conditions worked better and were therefore retained for 11.2 kb fragment. PrimeSTAR GXL has already been reported as the best-performing long-range DNA polymerase in comparison to five other DNA polymerases (22), specifically for the purpose of obtaining long PCR products for sequencing on MiSeq instrument. Even though a previous publication (22) used different targets for long-range PCR, our study corroborated the best performance of PrimeSTAR GXL, while also expanding its application to long-range PCR of mtDNA amplicons for MPS. Additionally, PrimeSTAR GXL provided accurate, repeatable, and reproducible results in our previous study (8), thus confirming its reliability for long-range PCR purposes.

Prior to testing the limited-cycle PCR test, we noticed that libraries showing low-quantity electropherograms on LabChip produced lower yield of generated sequencing data, which was not related to the loading concentration, thus causing lower cluster density. Moreover, with unmodified number of cycles some libraries even failed to achieve sufficient quantity for sequencing. As limited-cycle PCR also serves as library amplification step (besides the addition of index-adapters), we decided to increase the number of cycles in order to ensure sufficient quantity and better fragment distribution of libraries on LabChip. However, it was necessary to exclude the possibility that prolonged amplification of indexed libraries affected sequencing results in any way, for example, by elevating the level of noise or introducing sequence errors – all of which would interfere with the analysis and interpretation of results. Increasing amplification cycles produced larger quantity of libraries, which we believe also positively affected other quality metrics in sequencing runs. Since the modified conditions of limited-cycle PCR did not affect the level of noise or variant calling at the established analysis and interpretation thresholds, they were applied henceforth in our sequencing runs.

Sequencing metrics parameters (cluster density, clusters passing filter, quality of bases, etc) mostly depend on the loading concentration of pooled libraries, which is, in turn, mostly influenced by the accuracy of library quantification (23), thus not directly dependent upon the normalization method. However, the chosen method of library normalization may greatly impact the proportion of reads per sample (“% reads identified”), in the sense of great or poor uniformity among samples. Naturally, greater uniformity means a more even distribution of reads per sample, which is important in achieving sufficient read depth across the sequenced targets. Regions that receive very few or no reads make detection and interpretation of variants in those regions increasingly difficult, and would eventually require repeated library preparation and sequencing, which is not cost-effective. Our observation that % reads identified are more variable in bead-based normalization agrees with previously reported observations (4), and is likely caused by the sensitivity of magnetic beads to numerous handling steps (dependent on accuracy, precision, speed, and dexterity of particular analyst). Even though normalization beads are included in Nextera XT library preparation kit (ie, no additional reagent expenses), LabChip *cum* individual dilution involves fewer handling steps and requires less hands-on time. The potential for cross-contamination is thus diminished, though, at this stage, it is not critical, since all samples are already dual-indexed and would easily be resolved bioinformatically after sequencing. Considering negligible financial difference between the two methods, we find LabChip-based approach handier and less contamination-prone. Most importantly, it enables visual inspection of library fragments before sequencing, thus identifying libraries unsuitable for sequencing and reducing the cost of sequencing. Moreover, it provides slightly more uniform distribution of reads, which is important to avoid low-read-depth regions.

A limitation of this study is the number of samples included in experiments for optimizing target-enrichment and limited-cycle PCR. For practical and financial reasons, optimization experiments were performed in parallel to mtDNA population study (unpublished data), so there were not many additional samples that were repeatedly sequenced and could be included herein. By increasing the number of samples included in experiments, the correlations would possibly be clearer and achieve statistical significance. However, even with these shortcomings, it is still a valuable addition to our MPS workflow.

In conclusion, we optimized Illumina® whole mtDNA MPS assay on MiSeq FGx^TM^ instrument. Of the three tested high-fidelity long-range DNA polymerases, TaKaRa PrimeSTAR® GXL performed best in mtDNA enrichment from reference samples of buccal swabs and blood samples. Increment of amplification cycles to 15 (limited-cycle PCR step) did not affect sequencing noise in any way that would interfere with variant calling and interpretation, producing consistent and reproducible results. Furthermore, it increased the number of sequenceable libraries, with positive impact downstream on cluster density and average read depth per sample. In comparison to bead-based normalization, individual library normalization combined with quantification on LabChip® enabled more efficient sample processing and allowed visualization of fragment distribution within libraries. The latter is a particularly useful and cost-saving step for quality assurance prior to sequencing. Additionally, LabChip®-based approach provided more uniform distribution of reads per library, which is particularly important for detection of minor variants (eg, mitochondrial heteroplasmy). By achieving better uniformity of read distribution among samples, the chance of losing sequence information from regions with very few or no reads is greatly diminished. Altogether, optimizations reported herein surpass the solely intra-laboratory usage, and can be employed by other groups pursuing broad variety of PCR-based MPS workflows.
