# Supplementary material for: Human whole mitochondrial genome sequencing and analysis: optimization of the experimental workflow
Source: Croat Med J. 2022 Jun;63(3):224–30. doi: 10.3325/cmj.2022.63.224 (PMC9284014; doi:10.3325/cmj.2022.63.224)
Supplement: Supplementary Figure 1 [file CroatMedJ_63_s004.pdf]

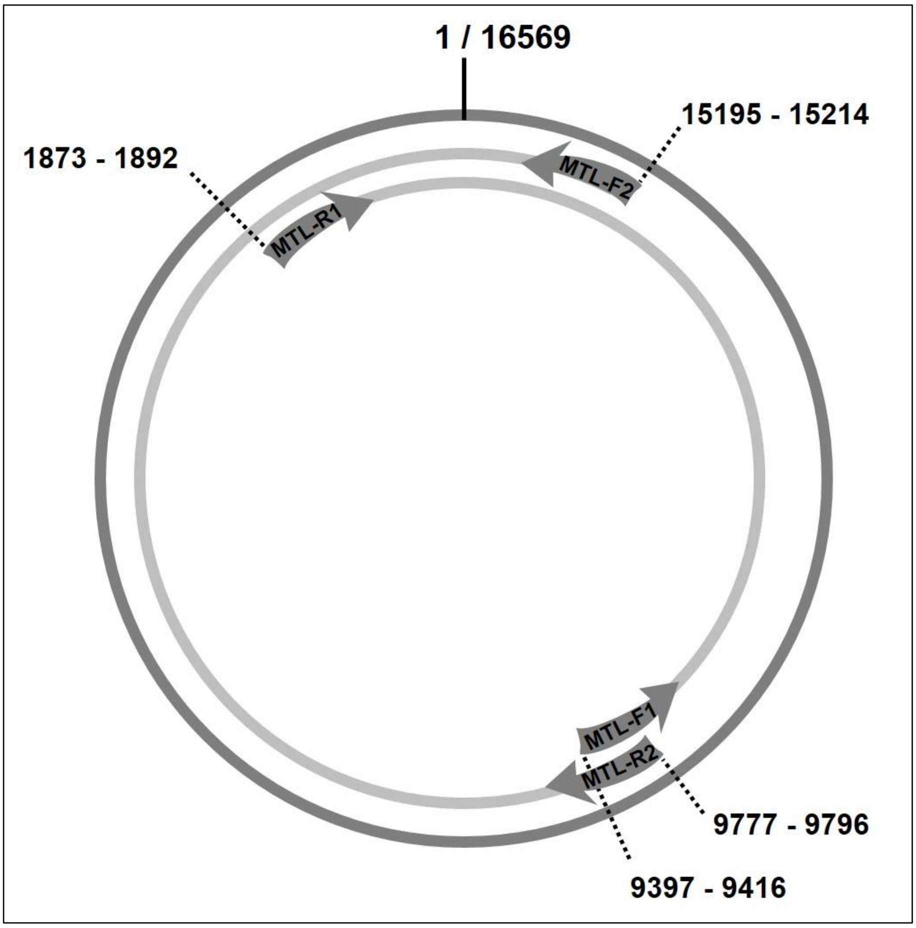

Supplementary Figure 1. Long-range PCR strategy employed by Illumina® Human mtDNA Genome assay*. Two pairs of primers are used to amplify human mitochondrial DNA in two large fragments: 9.1 kb (primers MTL-F1 and MTL-R1) and 11.2 kb (primers MTL-F2 and MTL-R2)†. Orientation, as well as start and end coordinates, are designated in the diagram for each primer.

* Illumina. Protocol: Human mtDNA Genome for the Illumina Sequencing Platform. 2016.
† Primer sequences:

MTL-F1: 5'- AAA GCA CAT ACC AAG GCC AC -3'
MTL-F2: 5'- TAT CCG CCA TCC CAT ACA TT -3'
MTL-R1: 5'- TTG GCT CTC CTT GCA AAG TT -3'
MTL-R2: 5'- AAT GTT GAG CCG TAG ATG CC -3'
