# Supplementary material for: Human whole mitochondrial genome sequencing and analysis: optimization of the experimental workflow
Source: Croat Med J. 2022 Jun;63(3):224–30. doi: 10.3325/cmj.2022.63.224 (PMC9284014; doi:10.3325/cmj.2022.63.224)
Supplement: Supplementary Figure 2 [file CroatMedJ_63_s005.pdf]

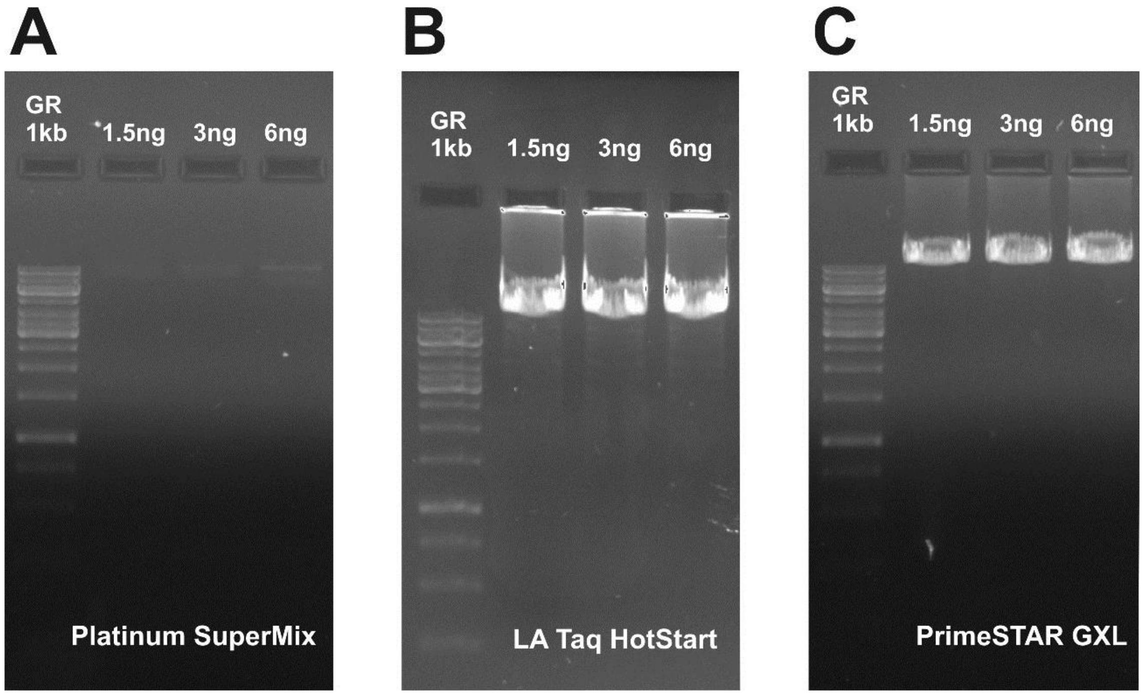

Supplementary Figure 2. In the first stage of DNA polymerase testing, identical buccal swab sample was used in all reactions (designated MW-002), with input of 1.5, 3 and 6 ng of genomic DNA. Mitochondrial DNA (mtDNA) fragment 11.2 kb was amplified at 30 cycles with all three DNA polymerases tested: Platinum™ PCR SuperMix High Fidelity (**A**), LA Taq® Hot Start polymerase (**B**), and PrimeSTAR® GXL polymerase (**C**). PCR products were visualized on 1% agarose gels beside GeneRuler 1 kb DNA ladder, where largest fragment size equals 10 kb (band quantity of approximately 15 ng of DNA, derived from product information sheet).
