# Supplementary material for: Human whole mitochondrial genome sequencing and analysis: optimization of the experimental workflow
Source: Croat Med J. 2022 Jun;63(3):224–30. doi: 10.3325/cmj.2022.63.224 (PMC9284014; doi:10.3325/cmj.2022.63.224)
Supplement: Supplementary Figure 3 [file CroatMedJ_63_s006.pdf]

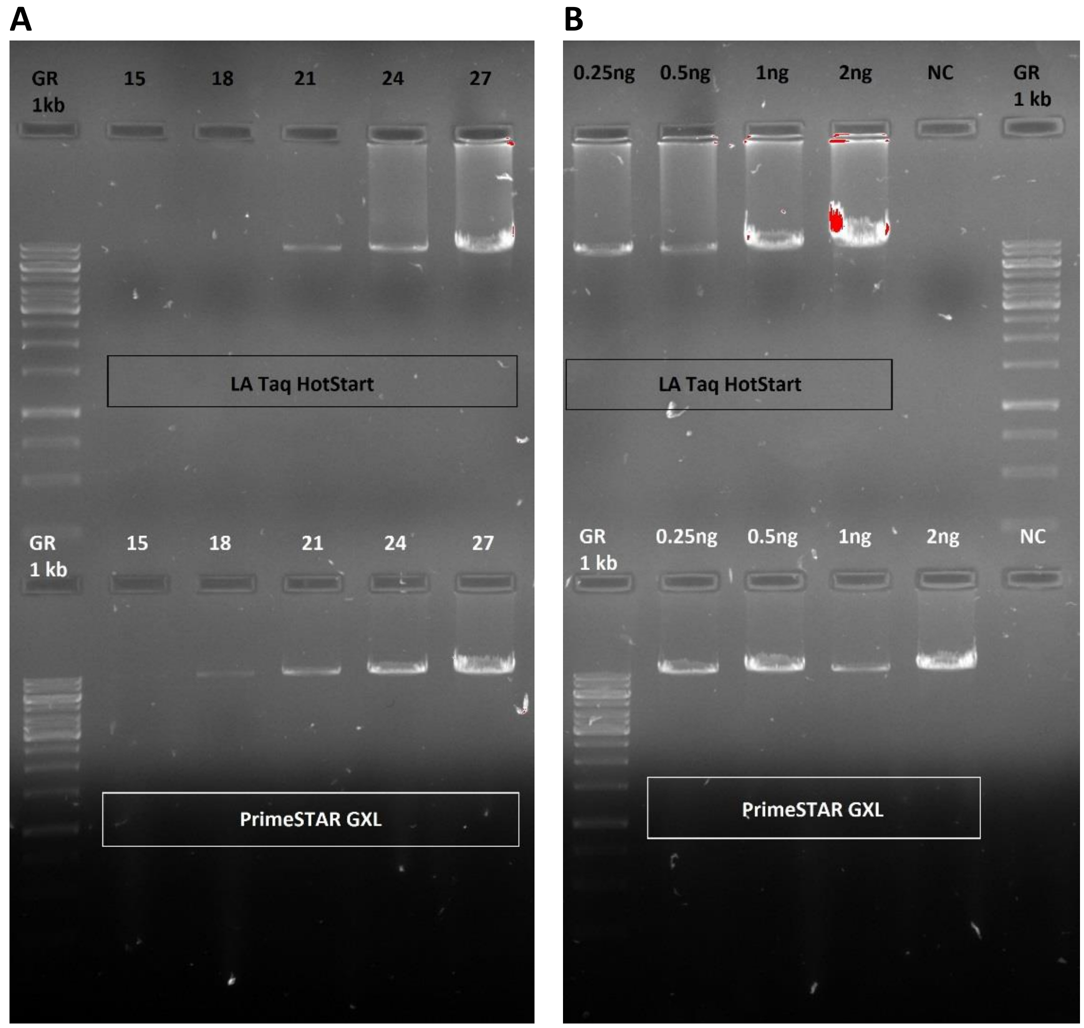

Supplementary Figure 3. In the second stage of DNA polymerase testing, 11.2 kb mtDNA fragment was amplified from the same buccal swab sample (designated MW-118) for both DNA polymerases, LA Taq® Hot Start and PrimeSTAR® GXL. Number of amplification cycles was tested (**A**), as well as input amount of genomic DNA (**B**). In **A**, input was 2 ng per 12.5 µL in all reactions, while in **B**, all reactions were performed with 30 cycles amplification. PCR products were visualized on 1% agarose gels beside GeneRuler 1 kb DNA ladder, where largest fragment size equals 10 kb (band quantity of approximately 15 ng of DNA, derived from product information sheet). NC = negative amplification control.
