# Supplementary material for: Human whole mitochondrial genome sequencing and analysis: optimization of the experimental workflow
Source: Croat Med J. 2022 Jun;63(3):224–30. doi: 10.3325/cmj.2022.63.224 (PMC9284014; doi:10.3325/cmj.2022.63.224)
Supplement: Supplementary Figure 4 [file CroatMedJ_63_s007.pdf]

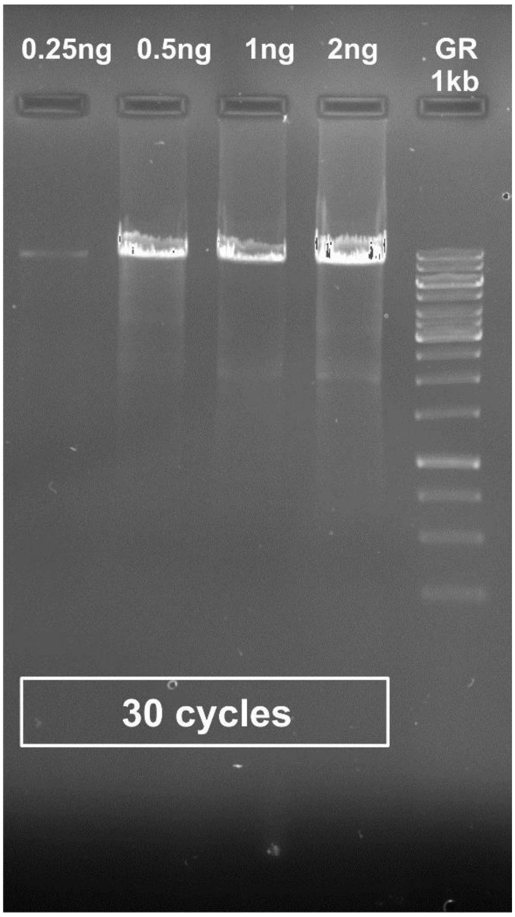

Supplementary Figure 4. In the third stage of DNA polymerase testing, where only PrimeSTAR® GXL was used, 9.1 kb mtDNA fragment was amplified from the same buccal swab sample (designated MW-118) in all reactions. Annealing temperature was set to 55°C. PCR products were visualized on 1% agarose gels beside GeneRuler 1 kb DNA ladder, where largest fragment size equals 10 kb (band quantity of approximately 15 ng of DNA, derived from product information sheet).
