# Supplementary material for: Human whole mitochondrial genome sequencing and analysis: optimization of the experimental workflow
Source: Croat Med J. 2022 Jun;63(3):224–30. doi: 10.3325/cmj.2022.63.224 (PMC9284014; doi:10.3325/cmj.2022.63.224)
Supplement: Supplementary Figure 5 [file CroatMedJ_63_s008.pdf]

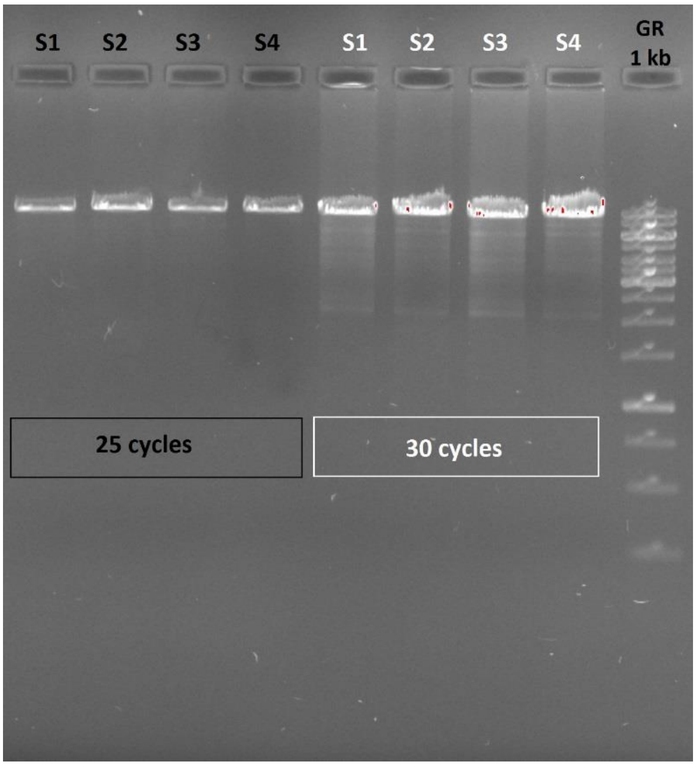

Supplementary Figure 5. In the fourth stage of DNA polymerase testing, only PrimeSTAR® GXL polymerase was used, with annealing temperature set to 60°C, in reactions with 25 and 30 amplification cycles. mtDNA fragment of 9.1 kb was amplified from 1 ng of genomic DNA of buccal swabs and blood samples from two persons (S1, S2 = samples of buccal epithelia and blood, respectively, from MW-020; S3, S4 = samples of buccal epithelia and blood, respectively, from MW-002). PCR products were visualized on 1% agarose gels beside GeneRuler 1 kb DNA ladder, where largest fragment size equals 10 kb (band quantity of approximately 15 ng of DNA, derived from product information sheet).

.
