# Supplementary material for: Human whole mitochondrial genome sequencing and analysis: optimization of the experimental workflow
Source: Croat Med J. 2022 Jun;63(3):224–30. doi: 10.3325/cmj.2022.63.224 (PMC9284014; doi:10.3325/cmj.2022.63.224)
Supplement: Supplementary Figure 6 [file CroatMedJ_63_s009.pdf]

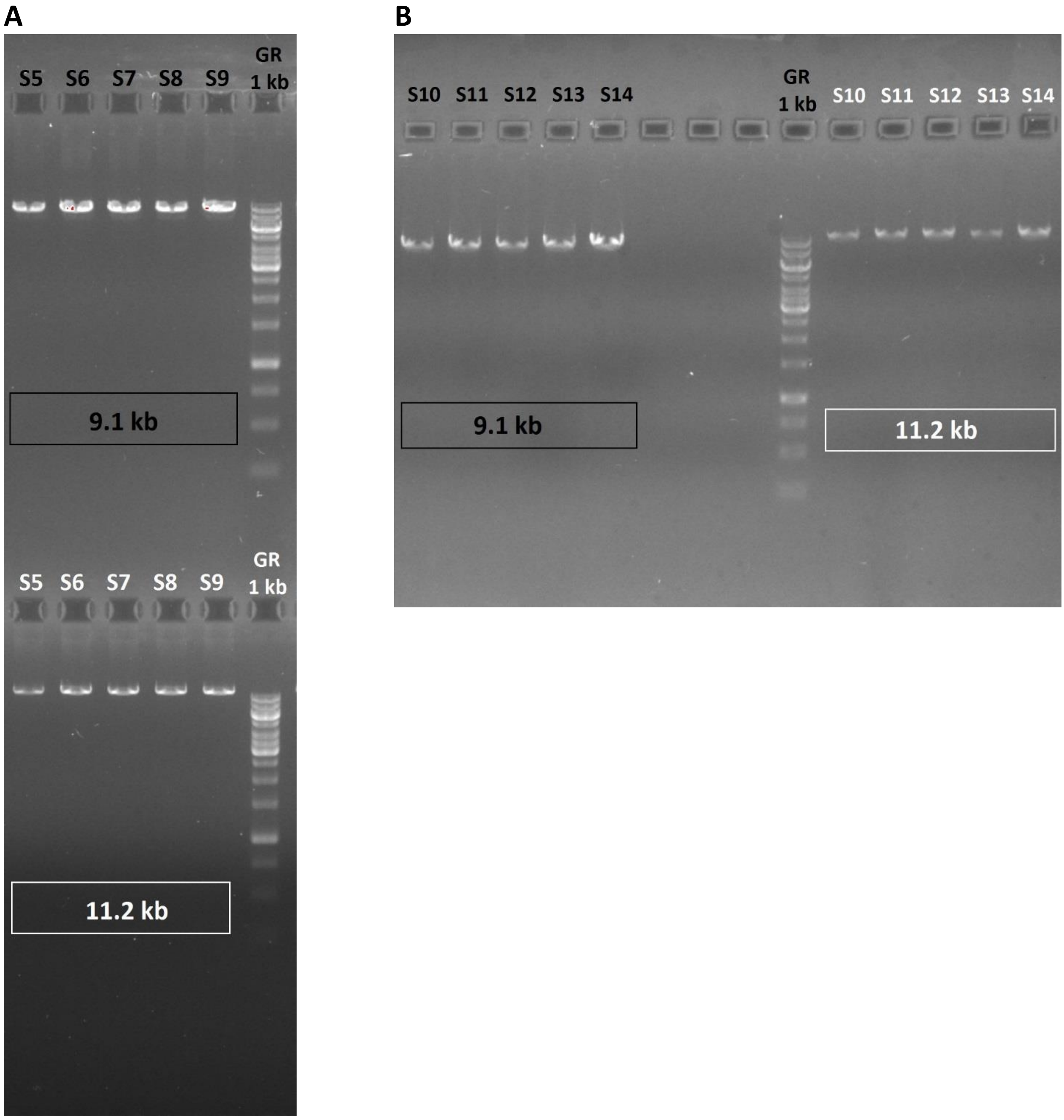

Supplementary Figure 6. Final optimized conditions for amplification of human mtDNA with PrimeSTAR® GXL polymerase resulted in clear, specific bands, as visualized on 1% agarose gels beside GeneRuler 1 kb DNA ladder, where largest fragment size equals 10 kb (band quantity of approximately 15 ng of DNA, derived from product information sheet). Amplification was successful in samples of five different persons (S5 – S9 = buccal epithelia samples, and S10 – S14 = blood samples of persons designated MW-012, MW-026, MW-065, MW-067 and MW-073) for both mtDNA fragments, 9.1 kb and 11.2 kb, and for both sample types (**A** - buccal swabs, **B** - and blood samples).
