# Supplementary material for: Human whole mitochondrial genome sequencing and analysis: optimization of the experimental workflow
Source: Croat Med J. 2022 Jun;63(3):224–30. doi: 10.3325/cmj.2022.63.224 (PMC9284014; doi:10.3325/cmj.2022.63.224)

Supplementary Figure 7. Distribution of fragments in ten libraries amplified with 12 cycles and 15 cycles in limited-cycle PCR step of Nextera® XT library preparation. Electropherograms produced by LabChip® DNA High Sensitivity Assay on LabChip® GX Touch HT.

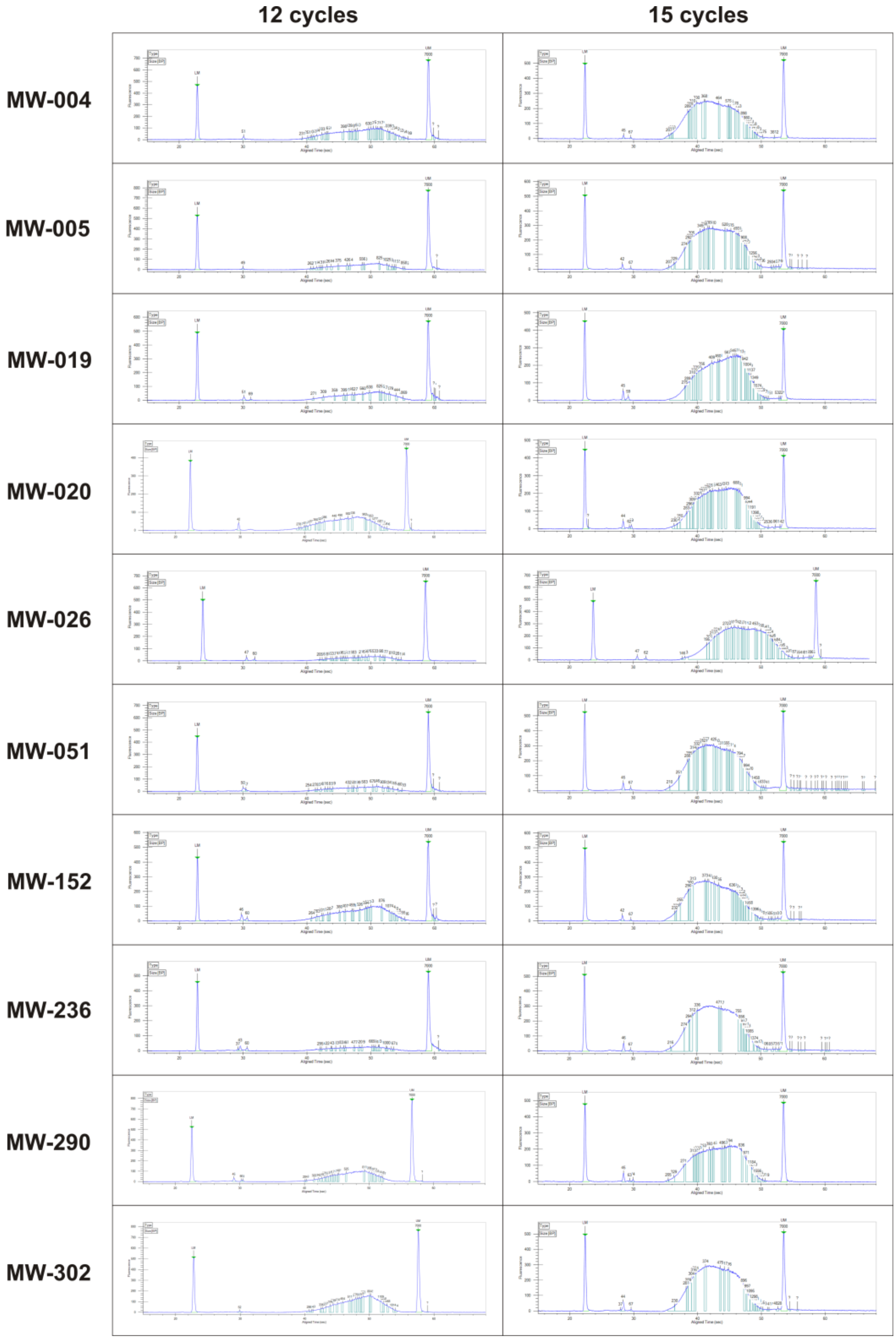

Supplement: Supplementary Figure 7 [file CroatMedJ_63_s010.pdf]
