# Supplementary material for: Human whole mitochondrial genome sequencing and analysis: optimization of the experimental workflow
Source: Croat Med J. 2022 Jun;63(3):224–30. doi: 10.3325/cmj.2022.63.224 (PMC9284014; doi:10.3325/cmj.2022.63.224)

Supplementary Figure 8. Distribution of "% reads identified" per sample for sequencing runs. Libraries in runs 1-3 underwent normalization with magnetic beads included in Illumina® Nextera® XT Library Prep Kit, while libraries in runs 4-6 were normalized individually according to molarities obtained by LabChip® DNA High Sensitivity Assay.

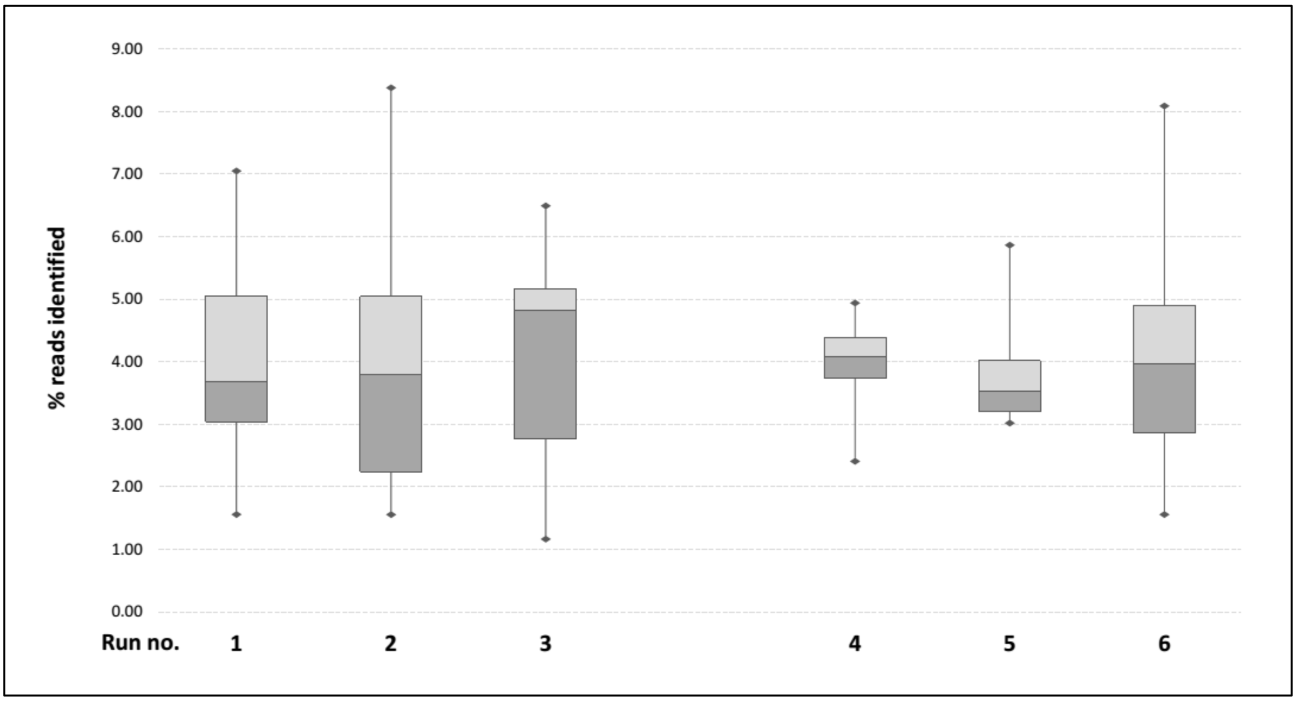

Supplement: Supplementary Figure 8 [file CroatMedJ_63_s011.pdf]
