# Supplementary material for: Human whole mitochondrial genome sequencing and analysis: optimization of the experimental workflow
Source: Croat Med J. 2022 Jun;63(3):224–30. doi: 10.3325/cmj.2022.63.224 (PMC9284014; doi:10.3325/cmj.2022.63.224)
Supplement: Supplementary Table 1 [file CroatMedJ_63_s012.pdf]

Supplementary Table 1. Gel-electrophoresis settings for evaluation of long-range PCR products.

**1% agarose gel**	Agarose – BioReagent, for molecular biology, low EEO (Sigma-Aldrich, St. Loius, MO, USA)	0.6 g
	TAE buffer (1 x)	60 mL
**Gel staining**	Midori Green Advanced DNA Stain (Nippon Genetics Europe GmbH, Düren, Germany) - added directly into gel, prior to casting	1 µL
**PCR products preparation**	4 µL PCR product<br>+ 1 µL DNA Loading Dye 6X (Thermo Fisher Scientific, Waltham, MA, USA)	
**Marker**	GeneRuler 1kb DNA ladder (Thermo Fisher Scientific, Waltham, MA, USA)	2.5 µL
**Instruments**	SubCell® GT system (Bio-Rad, Hercules, CA, USA)	
**Conditions**	80 V, 45 min	
**Visualization**	GelDoc™ system and Image Lab™ software (Bio-Rad, Hercules, CA, USA)	
