# Supplementary material for: Human whole mitochondrial genome sequencing and analysis: optimization of the experimental workflow
Source: Croat Med J. 2022 Jun;63(3):224–30. doi: 10.3325/cmj.2022.63.224 (PMC9284014; doi:10.3325/cmj.2022.63.224)
Supplement: Supplementary Table 2 [file CroatMedJ_63_s013.pdf]

Supplementary Table 2. Point heteroplasmies (PHP) detected in samples used for comparison of libraries produced by 12-cycle and 15-cycle limited-cycle PCR during Nextera® XT library preparation.

Sample ID	PHP	Major/minor allele	Minor allele % 12 cycles	Minor allele % 15 cycles
**MW-004**	16519Y	T/C	4.4	4.5
**MW-019**	16294Y	C/T	3.2	3.7
**MW-020**	152Y	T/C	5.6	5.7
	9325Y	T/C	5.4	6.2
**MW-026**	9740Y	C/T	20.9	20.6
	14539R	A/G	29.4	31.0
	16093Y	C/T	14.9	14.1
**MW-152**	12285Y	T/C	21.6	23.3
**MW-236**	152Y	T/C	15.6	15.1
	9111Y	T/C	44.6	45.5
	16189Y	T/C	21.7	19.9
**MW-290**	6899R	G/A	5.3	5.7
