# Supplementary material for: Human whole mitochondrial genome sequencing and analysis: optimization of the experimental workflow
Source: Croat Med J. 2022 Jun;63(3):224–30. doi: 10.3325/cmj.2022.63.224 (PMC9284014; doi:10.3325/cmj.2022.63.224)
Supplement: Supplementary Table 3 [file CroatMedJ_63_s014.pdf]

Supplementary Table 3. Analysis of negative controls (NCs) prepared with 12-cycle and 15-cycle "limited-cycle" PCR (**A** and **B**, respectively). Numbers in square brackets designate the quantity of each NC type that was analysed (8 and 6 in total, for 12 cycles and 15 cycles, respectively). NC-EX = extraction negative control; NC-PCR = long-range mtDNA enrichment PCR negative control; NC-LIB = library preparation negative control; DP = read depth (number of reads); Min = minimum; Max = maximum; SD = standard deviation.

A

	NC-EX [4]	NC-PCR [2]	NC-LIB [2]	Cumulative NCs [8]
**Min DP**	2	2	2	2
**Max DP**	46	15	14	46
**Average DP**	4	4	4	4
**SD**	3	2	4	3
**Total positions**	19 986	13 245	12 124	45 355

B

	NC-EX [3]	NC-PCR [2]	NC-LIB [1]	Cumulative NCs [6]
**Min DP**	2	2	2	2
**Max DP**	26	19	17	26
**Average DP**	6	5	5	6
**SD**	4	3	3	3
**Total positions**	23 780	10 794	9 646	44 220
